# A Retrospective Review of Thiazolidinediones with Development of a Troglitazone Conversion Protocol

**DOI:** 10.1100/tsw.2003.37

**Published:** 2003-06-09

**Authors:** Cameron Lindsey, Maqual Graham, Julie McMurphy

**Affiliations:** University of Missouri-Kansas City School of Pharmacy, MO 64108, USA

**Keywords:** thiazolidinediones, guideline adherence, drug utilization review, Veterans Affairs Medical Center

## Abstract

The objective of this paper was (1) to assess compliance with the National Veterans Affairs Guidelines for the use of troglitazone and rosiglitazone and (2) to develop and implement a conversion protocol that allows effective management of patients receiving troglitazone. A retrospective chart review was conducted to assess adherence to guidelines for all patients receiving troglitazone and rosiglitazone at the medical center. Appropriateness of therapy through indication evaluation, safety through alanine aminotransferase (ALT) monitoring compliance, and efficacy through hemoglobin A_1c_ (HbA_1c_) changes were used to assess adherence. According to National Veterans Affairs (VA) Guidelines, 68% of troglitazone and 63% of rosiglitazone patients had an appropriate indication for the use of these agents. Baseline ALT levels were obtained in 40% of troglitazone and 71% of rosiglitazone patients. Full compliance with continual ALT monitoring was seen in 6 and 54% of patients, respectively. Goal HbA_1c_ was achieved in 57 and 29% of patients, respectively. Of the 33 patients receiving troglitazone, 19 were converted to rosiglitazone therapy; 11 were maintained on current regimens without troglitazone, and 3 were lost to follow up. Adherence to guidelines needs to be reinforced, in particular, compliance with ALT monitoring. However, there were no reported cases of hepatotoxicity in the patients reviewed. Many patients did not achieve a HbA_1c_

The thiazolidinediones (glitazones) are a class of antidiabetic agents that exert their effects by increasing peripheral insulin sensitivity. Troglitazone (Rezulin®, Parke-Davis Division of Warner-Lambert Co., Warner-Lambert, Morris Plains, NJ), the first available agent in the class, has been associated with 90 reported cases of hepatotoxicity, 63 of which resulted in death, during its first 3 years on the market since its U.S. release in March of 1997[1]. The Food and Drug Administration (FDA) in collaboration with the drug's manufacturer released new guidelines to ensure appropriate use in patients. It was suggested that liver function monitoring occur at baseline, then monthly for the first year of treatment or after any dosage change, then quarterly thereafter. In addition, the indication for the use of troglitazone as monotherapy was withdrawn and recommendations were made regarding appropriate responses to elevated alanine aminotransferase (ALT) levels[2]. With the subsequent development of two new drugs in this class with similar efficacy and safer profiles, the FDA requested troglitazone be removed from the market. Troglitazone was withdrawn from the U.S. market March 21, 2000[3].

Rosiglitazone (Avandia®, SmithKline Beecham, Philadelphia, PA), the second drug in the thiazolidinedione class, was approved in May 1999[1]. The manufacturer, SmithKline Beecham, has stated this drug does not have troglitazone's risk of hepatotoxicity. Many fear, however, that clinical trials did not have sufficient sample size or duration of therapy to evaluate this risk[4]. For this reason, the manufacturer and the FDA recommend monitoring ALT in patients receiving rosiglitazone at baseline, every other month for the first year of therapy, and periodically thereafter[5].

Early in 2000, there were two case reports of suspected rosiglitazone-induced, idiosyncratic, hepatocellular injury; one resulting in death[1,6]. It is not fully known, however, whether all other risks or causes of hepatotoxicity were excluded in these patients. The American Diabetes Association[7] made the following statement in March 2000: "At this time, the American Diabetes Association does not believe that the safety concerns regarding Rezulin® extend to the other two medications [rosiglitazone and pioglitazone (Actos®, Takeda / Lilly, Lincolnshire, IL)]. However, as with all diabetes medications, this class of drugs will need further monitoring and research to ensure its long-term safety."

The Veterans Health Administration's Pharmacy Benefits Management Strategic Healthcare Group (PBM SHG) is charged with developing guidelines for the pharmacologic management of common disease states treated within the VA system. The PBM SHG relies on evidence-based, multidisciplinary, nationally recognized consensus statements as models for VA guideline development[8].

The glitazone guidelines specifically state indications for their use, safety monitoring requirements, and efficacy outcomes for the appropriate management of diabetic patients. Patients should not receive glitazones as first-line therapy due to the unpredictable potential to cause hepatotoxicity. Additionally, patients in the VA system who are prescribed other antidiabetic agents meet guideline criteria for glitazone use if they have failed monotherapy with a sulfonylurea or metformin and have failed or have a contraindication to combination therapy with metformin and a sulfonylurea. VA patients who are insulin dependent must use insulin in doses of greater than 75 units/day, have a $HbA_{1c}$ greater than 9%, or exceed target $HbA_{1c}$ value by more than 1%. Therefore, for this review, goal $HbA_{1c}$ was considered achieved if it was less than 8%.

These guidelines also define monitoring requirements for patients receiving a glitazone. The guidelines concur with FDA recommendations for the monitoring of a patient's ALT. The PBM SHG also suggests monitoring total bilirubin in addition to ALT. As bilirubin monitoring has not been common practice at the medical center due to following FDA guidelines, compliance with bilirubin monitoring was not evaluated by this review. HbA1c evaluation should be assessed every 3 months per guideline recommendations[8].

## OBJECTIVES

The first objective of this drug utilization review was to assess compliance with recommended monitoring guidelines for the use of troglitazone and rosiglitazone at the medical center with respect to: (1) appropriateness of therapy, through indication review; (2) safety, through baseline and continual monitoring of ALT; and (3) efficacy, through evaluation of $HbA_{1c}$. The second objective was to develop and implement a protocol that will maintain safe and efficient management of drug for patients following the unavailability of troglitazone.

## METHODS

A report comprised of medical center patients receiving either troglitazone or rosiglitazone from January 1, 1999 to December 31, 1999 was generated. At the request of the Pharmacy and Therapeutics (P&T) Committee, a computerized chart review was completed on all patients currently receiving either of these medications. A patient's therapy was considered appropriate if it met guideline criteria for use as previously defined. Full compliance of ALT monitoring, in accordance with VA guideline recommendations, was considered met if a patient received the total number of laboratory tests appropriate for the length of medication exposure. A percent of this number was used to quantify the data for each patient. Efficacy was evaluated in two manners: (1) comparison of baseline and current $HbA_{1c}$ and (2) whether or not patient achieved $HbA_{1c}$ goal. The secondary outcome of weight change was evaluated through comparison of baseline to current patient weight. Diabetes and nutrition education were considered to have occurred if the patient completed an individual consultation or group session. Indicators for cautious use or for avoiding use of these agents were also assessed (Table 1). Additional secondary outcomes evaluated included continuation of therapy without reaching $HbA_{1c}$ goal, discontinuation of therapy due to lack of efficacy, an adverse drug reaction (ADR), or death.

**TABLE 1**
**Indicators for Cautious Use or Avoidance**

| Indicators for Cautious Use | Indicators for Avoiding Use |
|---|---|
| NYHA[a] Class III or IV cardiac status | Type 1 diabetes |
| Edema | Pregnancy or lactation |
| Decreased white blood cell count | Increased ALT[b] (>1.5xULN[c] for troglitazone, > 2.5xULN for rosiglitazone) |
| Decreased hemoglobin or hematocrit | History of allergic reaction or hypersensitivity to either agent |
| Mild ALT[b] elevation (1–2.5 times the ULN[c]) | Acute liver disease |

[a] NYHA Class III or IV = New York Heart Association classification for congestive heart failure.
[b] ALT = alanine aminotransferase.
[c] ULN = upper limit of normal.

Development of the troglitazone protocol occurred in three phases. First, all troglitazone patients were re-evaluated for appropriateness of therapy according to guideline criteria. If a patient did not meet criteria for rosiglitazone use, all other antidiabetic agents were optimized to reach goal $HbA_{1c}$. If the patient met the criteria for the use of rosiglitazone, a pharmacist contacted the patient by phone to discuss this therapy option. During this conversation, the patient was educated on the withdrawal of troglitazone from the U.S. market, the available options for therapy, and the guideline requirements for the use of rosiglitazone in the VA system. The patients who chose to initiate rosiglitazone underwent a 1-week troglitazone-free washout period. Baseline ALT and total bilirubin were drawn at the completion of the washout, and if within normal limits, the patient was initiated on rosiglitazone 4 mg daily or other dose at the request of the patient's primary care provider.

Mean and standard deviations were calculated for demographic data. A paired Student t test was used to compare $HbA_{1c}$ and weight changes from baseline. *A priori* level of significance was set at <0.05.

## RESULTS

### Demographics and Patient Characteristics

A total of 59 patients were reviewed, of which 35 were troglitazone patients and 24 were rosiglitazone patients. All patients reviewed were male, and the mean age fell within the sixth decade in both groups.

### Appropriateness of Therapy

Prior to their initiation, 11 (31%) troglitazone patients and 9 (38%) rosiglitazone patients had not met guideline criteria for the use of these agents. Of the patients on an oral antidiabetic regimen, 9 (26%) troglitazone patients and 7 (29%) rosiglitazone patients had not failed, nor did they have a contraindication to, combination therapy with a sulfonylurea and metformin. In the insulin-requiring patients, 2 (6%) troglitazone and 2 (8%) rosiglitazone patients were initiated on glitazone therapy without sufficient daily insulin doses or $HbA_{1c}$ elevations to meet VA guidelines.

### Safety

Baseline ALT monitoring occurred in 14 (40%) troglitazone and 17 (71%) rosiglitazone patients; 2 (6%) troglitazone and 13 (54%) rosiglitazone patients had full compliance with ALT monitoring (Table 2).

**TABLE 2**
**ALT Monitoring Compliance**

| Compliance | Troglitazone (n = 35), n(%) | Rosiglitazone (n = 24), n(%) |
|---|---|---|
| 100% | 2(6) | 13(54) |
| 75–99% | 9(26) | 6(25) |
| 50–74% | 8(23) | 2(8) |
| <50% or unknown | 16(46) | 3(13) |

## Efficacy and Therapy Outcome

Troglitazone patients, overall, had a statistically significant decrease in $HbA_{1c}$ (9.64% +/- 3.46 to 8.01% +/- 1.53, $p$ = 0.033). The $HbA_{1c}$ decrease in the rosiglitazone group, however, was not statistically significant (10.1% +/- 1.82 to 9.24% +/- 2.11, $p$ = 0.088); 20 (57%) troglitazone patients and 7 (29%) rosiglitazone patients achieved guideline defined $HbA_{1c}$ goal of <8%.

## Secondary Outcomes

A mean weight increase was seen in both groups. This increase was statistically significant in the troglitazone group (6.26 lbs +/- 10.21, $p$ = 0.003); however, it was not statistically significant in the rosiglitazone group (2.45 lbs +/- 3.48, $p$ = 0.06). Diabetes education and nutrition education were completed in 12 (34%) of the troglitazone and 15 (63%) of the rosiglitazone patients; 20 and 50% of the patients completed both areas of education, respectively. Nutrition education was also completed in 34% of the troglitazone and 63% of the rosiglitazone patients. One troglitazone patient had an indication for avoiding use of the agent, due to a history of hepatitis C. This patient, however, had no elevation of ALT. Ten (42%) rosiglitazone patients had an indicator for cautious use of the agent. Five (21%) had a decrease in hemoglobin or hematocrit, three (13%) had edema, two (8%) had New York Heart Association class III or IV congestive heart failure (CHF), one (4%) had a decreased white blood cell (WBC) count, and one (4%) had mild ALT elevation. Continuation of therapy with $HbA_{1c}$ greater than or equal to 8% was seen in 14 (40%) and 13 (54%) of troglitazone and rosiglitazone patients, respectively. No patients were discontinued from therapy for lack of efficacy. No troglitazone patient reported an ADR during therapy. ADRs were reported with two rosiglitazone patients. One patient experienced a decrease in hemoglobin and a second patient with CHF developed worsening edema. Therapy was discontinued in the CHF patient, with resolution of symptoms. Two patients receiving rosiglitazone died, however, the etiology was not medication related.

## Troglitazone Conversion Protocol

Of the 35 patients on troglitazone, 19 met criteria for the use of rosiglitazone and underwent the conversion to this medication as previously described. Three patients in this group were lost to follow up. One patient began receiving care outside the VA system; a second patient moved to another VA; and the third was unable to be contacted. The complete results of this conversion process will be published separately.

## DISCUSSION

As with any retrospective review, there are limitations to this evaluation. First, patient progress notes had to be relied on for data collection. Therefore, if there was no mention of a particular event within the patient's records, it was assumed the event did not occur. Secondly, 11 (31%) troglitazone patients and 1 (4%) rosiglitazone patient received the majority of their care through an outside provider. Therefore, the available information on these patients was very limited and compliance with laboratory monitoring could not be fully evaluated in these patients. The fact that there were more troglitazone patients with an outside provider may be the reason the troglitazone patient group had a lower compliance rate than the rosiglitazone group.

Because at least one-third of patients in either group received medication without an appropriate indication, the P&T Committee at KCVAMC now requires all patients prescribed a glitazone undergo an indication review and consultation by a pharmacist to ensure appropriate use.

Although the compliance with ALT monitoring was not as high as we had hoped in either treatment group, there were no cases of hepatotoxicity. When considering the improved compliance results in the rosiglitazone group compared to the troglitazone group, one needs to consider compliance with lab monitoring in relation to patient education and knowledge. Although there were some guidelines in place when troglitazone became a formulary agent in the KCVAMC system, they were not as stringent as those developed when rosiglitazone was added. The more stringent guidelines included more frequent patient notification of the need for lab draws. Secondly, due to the occurrences of hepatotoxicity with the use of troglitazone, providers seemed to be more conscious of the need for this monitoring during this time. Patients became more aware of the need for monitoring as they were informed through lay press regarding the risk of hepatotoxicity from these agents. These factors need to be considered highly probable causes for the higher compliance rate in the rosiglitazone group.

When evaluating therapeutic outcome of change in $HbA_{1c}$ for the rosiglitazone patients, it is important to note the timeframe during which the patients received this medication. Considering the time of this review, the longest duration of therapy in the group was 5 months, with the mode falling around 3.5 months. This shortened length of therapy may be responsible for absence of a statistically significant difference in the $HbA_{1c}$ in this group, as one usually expects to appreciate the full potential of change in $HbA_{1c}$ after 3 months. More importantly, these patients would not have undergone their first dosage titration to further lower their $HbA_{1c}$.

Although a significant weight increase was observed in the troglitazone group, $HbA_{1c}$ appears unaffected. More rosiglitazone patients received education on diabetes and nutrition, which could potentially decrease their $HbA_{1c}$ more than troglitazone patients. As rosiglitazone patients were not evaluated for a sufficient length of time, this benefit may not have been able to be realized.

As this review was done in a VA setting, the results might not be reproducible in another patient setting. Additionally, the fact there were no females and that the mean age was over 60 excludes a great number of patients who would potentially use a thiazolidinedione for diabetes control. The VA setting also, as discussed above, lends to tighter monitoring from guidelines than is often seen outside the VA system. For these reasons, these data might not extrapolate to the entire diabetic community.

## CONCLUSION

With drugs being approved by the FDA at a more rapid rate, it is important to monitor patients more closely on therapies with a presumed or known risk for the development of ADRs once therapies are marketed. Troglitazone and rosiglitazone are two such medications. Adherence to the guidelines for the use of the glitazones at the medical center needs to be enforced to meet this demand. Development of the protocol allowed for efficient conversion of the patients' diabetes regimen while safely identifying those that should remain on this class of medication.

## ACKNOWLEDGMENTS

The authors wish to express thanks to Chris Meier, PharmD and Ruth Noland, PharmD for their review and contributions the manuscript preparation.

No funds were accepted for this project and no conflicts of interest exist with any of the authors. This publication is the result of work supported with resources and the use of facilities at the Veterans Affairs Medical Center, Kansas City, Missouri.

## BIOSKETCHES

**Cameron Lindsey, PharmD**, is an Assistant Professor at the University of Missouri-Kansas City School of Pharmacy (UMKC). Dr. Lindsey received her Doctor of Pharmacy degree from UMKC. Following graduation, she completed her post-doctoral training in an ASHP accredited Pharmacy Practice Residency at the KCVAMC and secured her current position.

Dr. Lindsey has instituted clinical pharmacy services at the KCVAMC. Additional roles include co-directing the residency and participation in drug utilization evaluations at the local, VISN and PBM levels. Research interests include the areas of cardiovascular risk reduction, congestive heart failure, diabetes and lipid management.

**Maqual Graham, PharmD**, received her doctor of pharmacy degree from UMKC in 1994. Dr. Graham completed an ASHP accredited residency in Pharmacy Practice at the KCVAMC in 1995. She currently serves as a Clinical Pharmacy Specialist at the same institution and is also an Assistant Professor of Pharmacy Practice at the UMKC School of Pharmacy. She is the principle investigator for numerous projects that focus on cardiovascular risk reduction. maqual.graham@med.va.gov.

**Julie McMurphy, PharmD,** received her doctor of pharmacy degree from UMKC in 1999. Dr. McMurphy completed an ASHP accredited residency in Pharmacy Practice at the KCVAMC in 2000. She received her Certified Diabetes Education credentials in 2002. She currently serves as Coordinator of Clinical Pharmacy Services at Bothwell Regional Health Center in Sedalia, MO and is an Adjunct Clinical Professor of Pharmacy Practice at the UMKC School of Pharmacy. jmcmurphy98@yahoo.com.
